# Arterial Spin Labeling MRI in Alzheimer's Disease: A Systematic Review of Cerebral Perfusion Biomarkers

**DOI:** 10.1111/jon.70035

**Published:** 2025-03-15

**Authors:** Caterina Bernetti, Valerio D'Andrea, Andrea Buoso, Ilenia Barbalace, Federico Greco, Fabio Pilato, Rosalinda Calandrelli, Vincenzo Di Lazzaro, Bruno Beomonte Zobel, Carlo A. Mallio

**Affiliations:** ^1^ Fondazione Policlinico Universitario Campus Bio‐Medico Rome Italy; ^2^ Research Unit of Radiology, Department of Medicine and Surgery Università Campus Bio‐Medico di Roma Rome Italy; ^3^ Department of Radiology Cittadella della Salute Azienda Sanitaria Locale di Lecce Lecce Italy; ^4^ Unit of Neurology Fondazione Policlinico Universitario Campus Bio‐Medico Roma Italy; ^5^ Radiology and Neuroradiology Unit, Department of Imaging, Radiation Therapy and Hematology, Università Cattolica del Sacro Cuore Fondazione Policlinico Universitario Agostino Gemelli—IRCCS Roma Italy

**Keywords:** Alzheimer's disease, arterial spin labeling (ASL), cerebral perfusion, MRI, systematic review

## Abstract

**Background:**

Alzheimer's disease (AD) is a leading cause of dementia. Arterial spin labeling (ASL) MRI, particularly at 3 Tesla (3T), offers a noninvasive method to assess cerebral blood flow alterations, which are believed to be early indicators of AD.

**Purpose:**

The purpose of this study is to evaluate the utility of 3T ASL MRI in identifying cerebral perfusion biomarkers for the diagnosis and management of AD, assess its prognostic value, and compare it to other imaging modalities, such as PET.

**Data Sources:**

A systematic literature search was conducted following the Preferred Reporting Items for Systematic Reviews and Meta‐Analyses guidelines across PubMed, Cochrane Library, and Scopus using keywords related to “ASL,” “3T MRI,” and “AD.”

**Study Selection:**

Studies were included if they used 3T ASL MRI to investigate CBF in AD. Reviews, preclinical studies, case reports, studies lacking 3T ASL MRI, or those focusing on other dementias or mild cognitive impairment without an AD comparison were excluded. Data extracted included study design, sample characteristics, imaging techniques, parameters measured, and outcomes. A qualitative synthesis of findings highlights CBF patterns and biomarkers associated with AD.

**Results:**

Findings demonstrated hypoperfusion in the hippocampus, precuneus, and posterior cingulate cortex, distinguishing AD from normal aging and other forms of dementia. CBF patterns are often correlated with the severity and progression of cognitive impairment. ASL MRI at 3T demonstrated diagnostic accuracy comparable to that of PET while being noninvasive and radiation free.

**Conclusion:**

ASL MRI at 3T could be a valuable tool for the early diagnosis and monitoring of AD. Its noninvasive nature makes it ideal for repeated measures and longitudinal studies. Further research should focus on standardizing protocols and validating their use in larger populations.

## 1 Introduction

### 1.1 Rationale

Alzheimer's disease (AD) is a neurodegenerative disorder characterized by a gradual progression of symptoms such as memory loss, cognitive decline, and behavioral changes, and it is the most common cause of dementia [1]. Its prevalence grew along with the aging of the global population, becoming one of the major public health challenges, right after cardiovascular and cerebrovascular diseases and cancer. Despite advances in understanding the pathological mechanisms underlying AD, including the accumulation of amyloid plaques and tau tangles, effective early diagnostic tools remain elusive [2]. Cerebral blood flow (CBF) alterations are widely believed to represent early events in the pathogenesis of AD, preceding significant structural changes in the brain [3, 4]. Hence, the identification of CBF biomarkers could be crucial for early diagnosis, disease progression monitoring, and therapeutic intervention evaluation.

Recent literature has emphasized the significance of arterial spin labeling (ASL) in AD studies [5, 6]. This noninvasive imaging technique—promising among MRI‐based perfusion biomarkers—has emerged as a valuable tool for the detection of CBF abnormalities in patients with AD and mild cognitive impairment (MCI) and could aid in early diagnosis, disease progression monitoring, risk stratification, and the evaluation of early therapeutic interventions [5, 6, 7, 8].

The diagnostic and prognostic utility of ASL MRI biomarkers, as well as their ability to differentiate AD from other forms of dementia and normal aging, requires further elucidation. In particular, understanding the regional perfusion patterns associated with AD and their relationship to disease severity and cognitive decline is essential for developing targeted interventions.

Previous studies using ASL in patients with AD have demonstrated significant CBF alterations in critical brain regions, such as the hippocampus, precuneus, posterior cingulate cortex (PCC), and temporoparietal areas [1]. Hypoperfusion in these areas is a consistent finding in ASL MRI studies of AD patients, distinguishing them from those with normal aging and other forms of dementia [9]. Compared to other imaging modalities, such as PET and SPECT, ASL‐MRI stands out for its noninvasiveness, lack of ionizing radiation, and absence of need for intravenous contrast agents or radioactive isotopes, achieving, however, comparable sensitivity and specificity in identifying AD [5, 7].

Furthermore, ASL MRI at 3 Tesla (T) could offer several advantages in the study of AD. In particular, the higher magnetic field strength improves the signal‐to‐noise ratio and spatial resolution, enabling more precise detection of CBF changes in the aforementioned brain regions.

### 1.2 Objective

This systematic review's objective is to evaluate the utility of 3T ASL MRI in the identification of significant cerebral perfusion biomarkers useful for the diagnosis and management of AD, assessing the prognostic value of this MRI sequence and also comparing it to other imaging modalities, such as PET.

## 2 Materials and Methods

### 2.1 Search Strategy

The systematic review was conducted according to the Preferred Reporting Items for Systematic Reviews and Meta‐Analyses guidelines. A systematic literature search was carried out in August 2023 using electronic databases: PubMed, Cochrane Library, and Scopus. There were no restrictions on the date range for the literature search.

The combination of keywords used to search the database was as follows: (“arterial spin labeling” OR “ASL”) AND (“MRI 3 T” OR “MRI 3 Tesla” OR “magnetic resonance imaging 3T” OR “magnetic resonance imaging 3 Tesla”) AND (“Alzheimer disease” OR “AD”).

### 2.2 Study Selection

**FIGURE 1:**
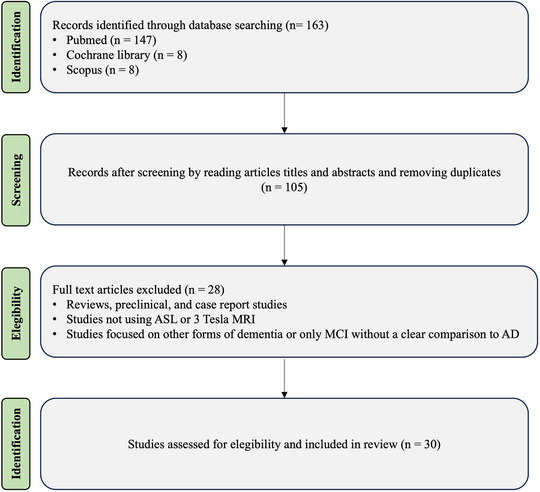
Preferred Reporting Items for Systematic Reviews and Meta‐Analyses diagram showing the flow of study selection through the phases of the review. AD, Alzheimer's disease; ASL, arterial spin labeling; MCI, mild cognitive impairment; *n*, number of studies.

After removing duplicates, the titles and abstracts of the remaining articles were screened to determine if they met the selection criteria. To further confirm eligibility, full texts of the remaining articles were screened using the following inclusion criteria: (1) studies exploiting 3T ASL MRI to identify cerebral perfusion biomarkers in individuals diagnosed with AD; (2) studies that recruited study participants who were 18 years of age or older; and (3) studies with a full text written in English. On the other hand, exclusion criteria were as follows: (1) reviews, preclinical studies, and case reports; (2) studies not using ASL; (3) studies not using 3T MRI; and (4) studies focusing on other forms of dementia or only on MCI without a clear comparison to AD. The complete study selection process is illustrated in Figure 1.

### 2.3 Data Extraction

The following data were systematically extracted: author and year of publication, study design, number of study participants, gender and age of the participants, imaging technique used, parameter measured, and study results.

## 3 Results

The systematic search resulted in a total of 163 articles. After removing duplicates, the remaining articles were screened for inclusion criteria based on title and abstract and, if necessary, full text. At the end of the complete screening, 30 articles were selected.

Study characteristics are reported in Table 1.

**TABLE 1:** Characteristics summary of the articles included in the paper.

Author Year Study design	Number of participants (*n*)	Imaging technique	Parameter measured	Study results
Nedelska et al. [18] 2018 Cross‐sectional	NC older adults: 76 Probable AD: 19 DLB: 76	3D pCASL 3T MRI 18F‐FDG‐PET 18F‐AV‐1451 tau PET	CBF Metabolism	AD: hypoperfusion in PCC; DLB: hypoperfusion in the precuneus and cuneus, PCC spared. Despite FDG‐PET being slightly more accurate, ASL still effectively differentiated between AD and DLB.
Thalman et al. [17] 2020 Cross‐sectional	DS: 34 (DSnon: 20, DSi: 7, probable AD: 7)	3D pASL 3T MRI	CBF	Significantly lower CBF in DS with probable AD, associated with worst cognitive impairment.
Collij et al. [31] 2016 Cross‐sectional	NC: 26 MCI: 60 (12 converted to AD) Probable AD: 100 SCD: 100	pCASL 3T MRI Machine learning	CBF	High accuracy in the automated classification of perfusion maps, distinguishing AD from other conditions, and predicting MCI to AD conversion.
Song et al. [2] 2023 Cross‐sectional	Probable MCI or AD: 23	pCASL 3T MRI PET (amyloid‐β tracer 11C‐Pittsburgh Compound B)	CBF Amyloid‐β accumulation Parasagittal dural space volume	Hypertrophy of the parasagittal space may be more closely related to cerebral Aβ retention in AD, rather than ChP perfusion or bulk CSF net flow.
Wang et al. [26] 2022 Cross‐sectional	NC: 33 AD: 45	pCASL 3T MRI T1‐mapping	CBF T1 values	Significantly lower CBF and higher T1 values in the right caudate nucleus and left hippocampus in AD patients, compared to the NC group. Higher diagnostic accuracy for AD combining pCASL with T1‐mapping.
Morgan et al. [12] 2021 Cross‐sectional	NC: 20 MCI: 45 AD: 13 SCD: 44	pCASL 3T MRI	CBF Spatial heterogeneity of CBF (ASL sCoV)	Increased spatial variation in CBF correlated with cognitive decline, suggesting sCoV as a marker for AD progression.
Zhang et al. [5] 2022 Cross‐sectional	NC: 50 AD: 53	pCASL 3T MRI DL	CBF DL‐based denoising	DL‐based denoising enhances sensitivity in detecting AD‐related hypoperfusion, particularly in the precuneus and parietal regions.
Li et al. [24] 2020 Cross‐sectional	NC: 19 MCI: 18 AD: 22 SCD: 25	pCASL 3T MRI QSM	CBF Iron deposition	Decreasing CBF and increasing iron deposition in critical brain regions in AD progression.
Chau et al. [21] 2020 Cross‐sectional	NC: 15 AD: 17 18: T2DM SCD: 8 VD: 12	pCASL 3T MRI 18‐F flutemetamol PET‐CT	CBF Amyloid burden	Similar CBF reductions in AD and T2DM, indicating possible shared cerebrovascular dysfunction.
Thomas et al. [23] 2019 Cross‐sectional	NC: 21 MCI: 20 AD: 19	pCASL 3T MRI 3D‐BRAVO 3T MRI	CBF Morphology	Combining entorhinal cortical atrophy scores with CBF measurements improved differentiating MCI from AD.
De Jong et al. [16] 2019 Clinical trial	58	ASL 3T MRI	CBF changes	Nilvadipine increased hippocampal CBF in AD patients, suggesting potential therapeutic benefits and the ability of ASL to follow up after treatment.
Huang et al. [9] 2019 Cross‐sectional	NC: 40 AD or MCI: 40	3D‐PCASL 3T SWI	CBF Microhemorrhages	AD patients showed reduced CBF and more microhemorrhages compared to MCI.
Shirzadi et al. [15] 2019 Cross‐sectional	NC: 41 MCI: 88 AD:30	Pulsed ASL 3T MRI	CBF Spatial heterogeneity of CBF (ASL sCoV)	Higher spatial heterogeneity in CBF distinguished AD from MCI and cognitively normal individuals.
Göttler et al. [27] 2019 Cross‐sectional	NC: 27 AD: 42	pASL 3T MRI BOLD PET/CT 18F‐FDG	CBF Functional connectivity Metabolism	BOLD‐FC, CBF, and glucose metabolism were reduced in precuneus parietal regions. BOLD‐FC reductions are aligned with hypoperfusion at ASL‐MRI, regardless of glucose hypometabolism at FDG‐PET.
Kaneta et al. [10] 2017 Cross‐sectional	AD or MCI: 41	3D PCASL 3T MRI SPECT (123I‐IMP)	CBF (with different PLD) Metabolism	Significant positive correlations between CBF, measured with both ASL‐MRI and SPECT, and cognition were found in the PCC and temporoparietal association cortices.
Leeuwis et al. [14] 2016 Cross‐sectional	MCI: 95 AD: 161 SCD: 143	pCASL 3T MRI	CBF Cognitive impairment	Lower CBF in the parietal and temporal regions was strongly associated with cognitive impairments in AD.
Liu et al. [28] 2015 Cross‐sectional	NC: 19 AD: 16	pCASL 3T MRI	CBF (with different PLD)	Shorter PLD improved the detection of hypoperfusion in AD, especially in the precuneus and PCC.
Steketee et al. [19] 2016 Cross‐sectional	NC: 47 AD: 13 FTD: 19	pCASL 3T MRI	CBF	ASL MRI differentiated AD from FTD by showing hypoperfusion in PCC in AD and in the anterior cingulate cortex in FTD.
Zou et al. [25] 2014 Cross‐sectional	NC: 20 AD: 20	pCASL 3T MRI (QUASAR) MRS	CBF MRS	Regional hypoperfusion and abnormal metabolic changes in specific AD‐related regions.
Benedictus et al. [4] 2014 Cross‐sectional	NC: 61 AD: 129	pCASL 3T MRI	CBF Brain volume WMH	Reduced brain volume and larger WMH associated with lower CBF in AD, indicating combined neurodegeneration and vascular dysfunction.
Ding et al. [13] 2014 Cross‐sectional	NC: 21 MCI: 17 AD: 24	pCASL 3T MRI (3D)	CBF	Hypoperfusion in the parietooccipital cortices in AD and hyperperfusion in the frontal regions in MCI, as early perfusion changes are detectable by ASL MRI.
Le Heron et al. [20] 2014 Cross‐sectional	NC: 37 AD: 17 PDD: 20	pCASL 3T MRI	CBF	Identified distinct perfusion patterns, with AD showing reduced perfusion in the medial temporal lobes and PDD in the right frontal cortex, aiding in differential diagnosis.
Mak et al. [11] 2014 Cross‐sectional	NC: 15 AD: 13	pCASL 3T MRI (QUASAR)	CBF Hippocampal volumetry	Combining hippocampal volumetry with ASL MRI significantly improved the diagnostic accuracy for differentiating AD from cognitively normal adults.
Kilroy et al. [30] 2014 Cross‐sectional	NC: 6 MCI: 6 AD: 1	pCASL 3T MRI (2D) GRASE H215O PET	CBF	3D GRASE pCASL MRI showed higher reliability and better agreement with 15O‐water PET compared to 2D EPI pCASL, making it a superior method for CBF measurement in AD.
Binnewijzend et al. [7] 2013 Cross‐sectional	MCI: 35 AD: 71 SCD: 73	pCASL 3T MRI (3D)	CBF	3D pCASL MRI effectively measures CBF reductions in AD and MCI, particularly in the precuneus and PCC, correlating with cognitive decline.
Mak et al. [3] 2012 Cross‐sectional	NC: 20 AD: 20	pCASL 3T MRI (QUASAR)	CBF aBV and aTT	Significant reductions in CBF and arterial blood volume, and prolonged arterial transit time in AD, correlating with cognitive impairment.
Dashjamts et al. [22] 2011 Cross‐sectional	NC: 23 AD: 23	ASL 3T MRI T1 structural MRI	CBF GM density	Combination of CBF measurement and morphological analysis based on VBM procedure was more effective in discriminating AD from NC than either method alone.
Xu et al. [29] 2010 Cross‐sectional	22	pCASL 3T MRI 15O‐water PET	CBF Metabolism	pCASL provides a reliable CBF measurement in young and elderly adults, converging with results obtained with 15O‐water PET perfusion.
Yoshiura et al. [8] 2009 Cross‐sectional	NC: 23 AD: 20	pCASL 3T MRI (QUASAR)	CBF	Comparisons of CBF maps revealed areas of significant hypoperfusion in AD patients in the bilateral precunei and PCC, suggesting ASL's role in discriminating AD individuals from normal subjects.
Alsop et al. [1] 2008 Cross‐sectional	NC: 16 AD: 22	ASL 3T MRI T1 structural MRI	CBF Brain volume	The hippocampus and other regions affected early in AD characterized by elevated atrophy‐corrected perfusion, suggesting compensatory or pathological elevation of neural activity or inflammation.

Abbreviations: 123I‐IMP, *N*‐isopropyl‐4‐[123I]iodoamphetamine; 18F‐AV‐1451, 18F‐flortaucipir (AV‐1451) tau PET; 3D pASL, 3D pulsed ASL; 3D pCASL, 3D pseudocontinuous ASL; aBV, arterial blood volume; AD, Alzheimer's disease; ASL, arterial spin labeling; aTT, arterial transit time; Aβ, amyloid‐β; BOLD‐FC, blood‐oxygen‐level‐dependent functional connectivity; BRAVO, brain volume; CBF, cerebral blood flow; ChP, choroid plexus; DL, deep learning; DLB, dementia with Lewy bodies; DS, Down syndrome; DSi, indeterminate dementia; DSnon, no dementia; EPI, echo‐planar imaging; FDG‐PET, fluorodeoxyglucose PET; FTD, frontotemporal dementia; GM, gray matter; GRASE, gradient and spin echo; MCI, mild cognitive impairment; MRS, magnetic resonance spectroscopy; NC, normal controls; PCC, posterior cingulate cortex; PDD, progressive supranuclear palsy; PLD, postlabeling delay; QSM, quantitative susceptibility mapping; QUASAR, QUAntitative Star labeling of Arterial Region; SCD, subjective cognitive decline; sCoV, spatial coefficient of variation; SWI, susceptibility‐weighted imaging; T, Tesla; T1, T1‐weighted; T2DM, type‐2 diabetes mellitus; VBM, voxel‐based morphometry; VD, vascular dementia; WHM, white matter hyperintensities.

### 3.1 Study Selection

**FIGURE 2:**
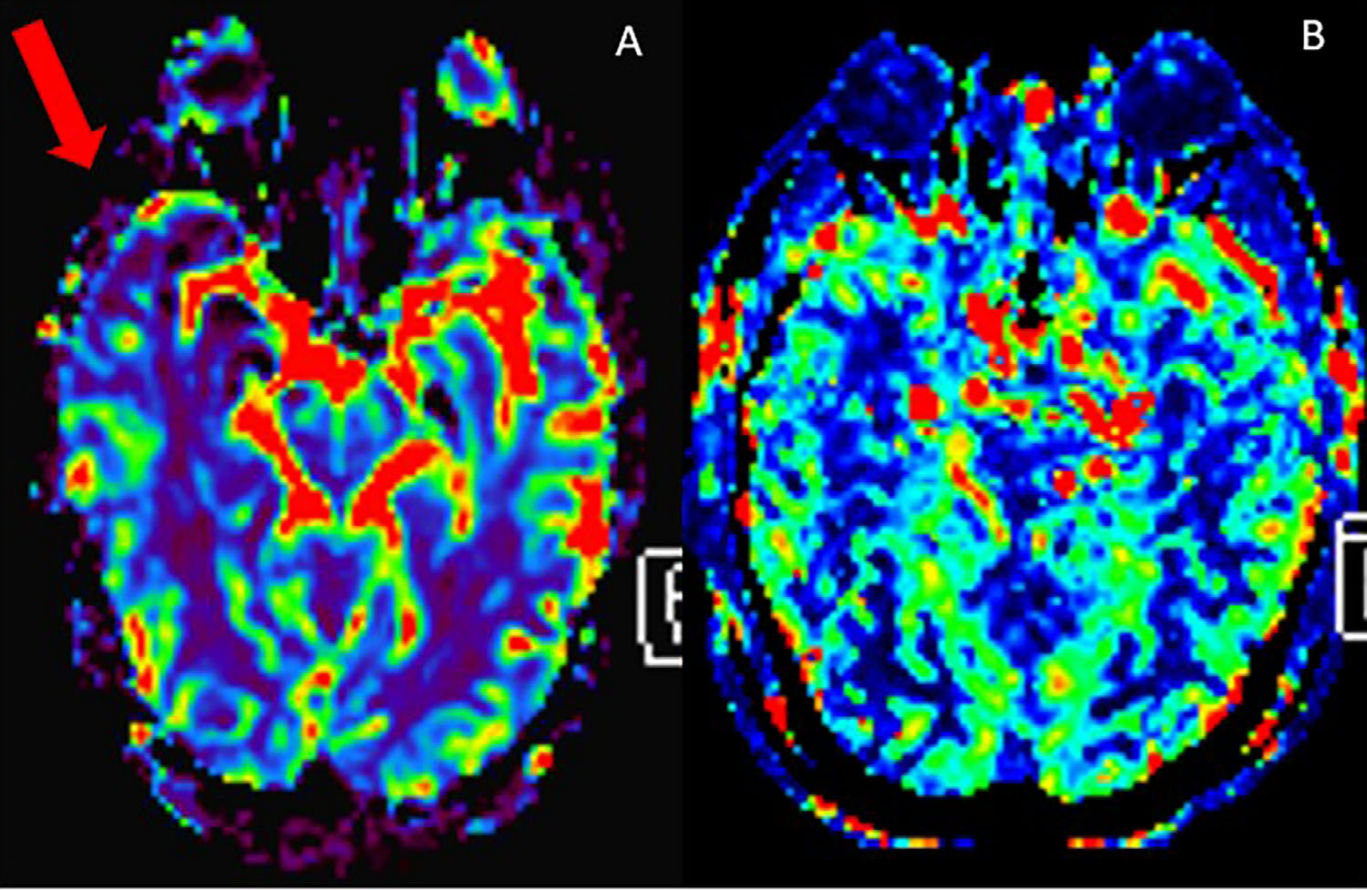
Color‐coded cerebral blood flow maps acquired from ASL images showing a reduction in perfusion (dark blue) in the parietal and temporal regions, more evident on the right side, in a 78‐year‐old patient with Alzheimer's disease (red arrow, A), compared to an 81‐year‐old cognitively unimpaired patient (B).

A total of 30 studies were included in this review, focusing on the use of 3T MRI ASL to assess CBF in patients with AD (Figure 2). The studies employed various designs, including clinical trials and observational studies. The studies predominantly utilized ASL sequences, comprising three‐dimensional pseudocontinuous ASL (3D‐pCASL), to measure CBF. Additionally, some studies incorporated other sequences, such as susceptibility‐weighted imaging (SWI), magnetic resonance spectroscopy (MRS), or voxel‐based morphometry, to further explore brain structure and function.

## 4 Results of the Studies and Discussion

### 4.1 Pattern of Hypoperfusion

Several studies using 3T ASL MRI have identified typical cerebral hemodynamic parameters useful for the diagnosis of AD, most commonly being hypoperfusion in the hippocampus, precuneus, and PCC. These regions are critically involved in memory and cognitive functions, which are typically impaired in AD.

Yoshiura et al. demonstrated that 3T ASL MRI effectively detects AD by identifying significant hypoperfusion in the precuneus and PCC, achieving high diagnostic accuracy with area under the curve (AUC) values ranging from 0.861 to 0.932 [8]. A reduced CBF in the PCC, as well as in temporoparietal regions, was also reported by Kaneta et al. [10].

Huang et al. conducted a study exploring the use of 3D‐pCASL and observed a decreased CBF in the hippocampus, frontal gray matter, and occipital gray matter in AD patients [9].

Mak et al. utilized QUAntitative Star labeling of Arterial Region (QUASAR) ASL at 3T to quantitatively assess cerebral hemodynamic parameters in AD patients. The results showed significant reductions in CBF in regions like the middle cingulate and PCC in comparison to cognitively normal elderly adults [11]. Additionally, arterial blood volume was lower and arterial transit time was prolonged in those specific regions, also indicating underlying vascular abnormalities in AD [11]. The identification of these hemodynamic hypoperfusion changes correlated with cognitive impairment supports the diagnostic and prognostic potential of 3T ASL MRI in AD.

However, an interesting result was reported by Alsop et al., who identified hippocampal hyperperfusion in AD using 3T ASL MRI after correcting for gray matter loss, suggesting increased blood flow despite significant atrophy [1]. This hyperperfusion may indicate compensatory neural activity or inflammation possibly present in early AD.

### 4.2 Prognostic Value

The prognostic utility of 3T ASL MRI in predicting AD progression is a significant focus in the literature. In fact, several studies have also found that 3T ASL MRI biomarkers are not only indicative of current cognitive status but also change significantly during progression from preclinical stages, such as MCI, to advanced AD [3].

Among these biomarkers, reduced CBF in the hippocampus, precuneus, and PCC is believed to be the most predictive of AD progression and clinical severity, correlating with rapid cognitive decline, worsening memory, and impaired executive function [12].

Ding et al. found a difference in hypoperfused regions between AD and MCI, with significant hypoperfusion in the parietooccipital cortices in AD and significant frontal hyperperfusion in MCI [13].

Leeuwis et al. also underlined the potential of ASL as an assessment tool for evaluating the severity of cognitive decline in AD patients, demonstrating a link between lower CBF in the hippocampus and PCC and impairments across several cognitive domains, including global cognition, executive function, and language [14].

Binnewijzend et al. showed that 3D pCASL MRI can differentiate between stages of cognitive decline in AD severity by detecting CBF reductions in the precuneus and PCC, which are more pronounced in AD compared to MCI [7].

Shirzadi et al. found that spatial heterogeneity in CBF, measured by the spatial coefficient of variation (sCoV) using 3T ASL MRI, effectively distinguished AD from MCI and healthy individuals, showing the greatest between‐group difference in the temporal lobe [15]. Also, Morgan et al. found that sCoV in CBF maps progressively increases with cognitive decline, from MCI to early AD, suggesting its potential role as a marker for tracking cognitive deterioration and predicting disease progression [12].

These findings underscore the value of these biomarkers for prognostic assessments, predicting future cognitive deterioration [1, 5, 7, 9, 14].

Furthermore, some studies investigated the effects of treatment on CBF in AD patients using 3T ASL MRI, discovering that therapeutic interventions were associated with changes in CBF, which indicates potential benefits in brain perfusion and suggests that 3T ASL MRI could be used to monitor treatment efficacy in AD patients. For instance, De Jong et al. showed that Nilvadipine treatment led to an increased hippocampal CBF in patients with mild to moderate AD, while global CBF remained stable, suggesting potential benefits in AD management [16].

### 4.3 Differentiating Patterns from Normal Aging and Other Dementias

AD's hypoperfusion patterns are distinct from those seen in other dementias and in normal aging. For instance, normal aging is associated with more uniform, less region‐specific reductions in perfusion, whereas hypoperfusion focused in the hippocampus, precuneus, and PCC is more typical in AD [9].

Thalman et al. found significantly lower CBF in individuals with Down syndrome who have AD compared to those with Down syndrome without dementia, with a strong association with more severe cognitive impairment, highlighting its potential as an early marker of AD in this population [17].

A study by Nedelska et al. found that AD patients exhibit significant hypoperfusion in the PCC, distinct from dementia with Lewy bodies (DLB), in which this region is relatively spared while the precuneus and cuneus show more pronounced hypoperfusion [18].

Steketee et al. demonstrated the ability of 3T ASL MRI to distinguish frontotemporal dementia by identifying hypoperfusion in the anterior cingulate cortex and AD with a more pronounced hypoperfusion in the PCC [19].

The ability of ASL MRI to differentiate AD from other dementias, such as Parkinson's disease dementia (PDD), was also explored by Le Heron et al., discovering that while both AD and PDD exhibit hypoperfusion in posterior brain regions, specific differences could be detected using ASL 3T MRI, aiding in differential diagnosis. In particular, AD showed relatively reduced perfusion in the medial temporal lobes, while PDD exhibited more pronounced hypoperfusion in the right frontal cortex [20].

A curious finding has, however, been described by Chau et al., who discovered a similar significant reduction in CBF in AD and type 2 diabetes mellitus, suggesting that both conditions may share underlying cerebrovascular dysfunctions [21].

### 4.4 Association with Structural Changes or Other MRI Biomarkers

Several studies have investigated the association of alteration in CBF with structural changes or other MRI biomarkers.

Dashjamts et al. demonstrated that combining 3T ASL MRI with morphological assessments significantly improves diagnostic accuracy for AD. In particular, the combined evaluation of ASL regional CBF and gray matter density analysis resulted in an AUC of 0.919, outperforming either method alone [22].

Similarly, Mak et al. demonstrated that combining hippocampal volumetry with CBF measurements significantly improved the accuracy of distinguishing AD patients from cognitively normal elderly adults, achieving high diagnostic accuracy with an AUC of 0.944 [4].

Also, Thomas et al. demonstrated that combining entorhinal cortical atrophy scores with CBF measurements in the precuneus and PCC significantly improves diagnostic accuracy for and the differentiation between MCI and AD [23].

Benedictus et al. found that in AD patients, lower CBF measurements are significantly associated with smaller brain volumes and larger white matter hyperintensities, suggesting that decreased CBF in AD could be influenced by both neurodegeneration and small vessel disease, highlighting the possible synergic impact of these pathologies on cerebral perfusion [4].

Furthermore, Huang et al. reported a notable increase in microhemorrhages detected via SWI, along with a reduction in CBF [9]. In their study, Li et al. found an inverse correlation between CBF, which decreases in AD, and higher iron deposition, particularly in the putamen and hippocampus. These changes may serve as biomarkers for monitoring the progression of AD [24].

In addition, Zou et al. demonstrated that using both ASL and MRS enhances the ability to detect and monitor AD by identifying significant reductions in CBF and associated abnormal metabolic changes in AD's critical brain regions, such as the PCC [25].

Song et al. demonstrated that parasagittal dural space (PSD) volume, particularly in the frontal and parietal subregions, was correlated with global amyloid‐β (Aβ) burden, suggesting that PSD hypertrophy may play a role in impaired CSF clearance, contributing to Aβ accumulation in AD [2].

The study by Wang et al. demonstrated that combining pCASL with T1‐mapping significantly improves diagnostic accuracy for AD compared to using either method alone [26].

Göttler et al. demonstrated a significant association between reduced BOLD functional connectivity in the posterior default mode network and decreased CBF in AD, suggesting that the impaired neural connectivity observed in AD is strongly linked to underlying vascular dysfunction and is mostly independent of glucose metabolism [27].

### 4.5 Diagnostic Accuracy Compared to Other Imaging Modalities

ASL MRI findings are generally consistent with those obtained from other techniques like PET or SPECT. In fact, several studies show that 3T ASL MRI achieves a sensitivity of 80%–90% and a specificity of 70%–85% in diagnosing AD, which is comparable to PET imaging, particularly with amyloid tracers [28].

Compared to PET, which is often more specific for AD due to amyloid imaging, ASL MRI provides a broader assessment of hemodynamic changes, which can be critical for understanding disease mechanisms beyond amyloid deposition [26].

Xu et al. evaluated the reliability and precision of pCASL at 3T in elderly subjects at risk for AD, comparing it with the gold‐standard 15O‐water PET. The results reported high reliability for pCASL across various brain regions, with strong agreement between pCASL and PET measurements, particularly in the PCC [29].

Kilroy et al. also reported a better correlation between 3D gradient and spin echo (GRASE) pCASL and the gold‐standard 15O‐water PET imaging [30]. Similarly, Nedelska et al.’s findings of hypoperfusion in the PCC in AD are consistent with the hypometabolism observed in these regions using FDG‐PET, supporting the role of ASL MRI as a noninvasive alternative to FDG‐PET in clinical practice to differentiate AD from DLB [18].

ASL MRI has several advantages over other imaging modalities, including its noninvasive nature, absence of radiation, possibility of repeated measures, and ability to provide quantitative CBF measurements, making it suitable for routine use and longitudinal studies [7, 11, 22]. MRI is also more cost‐effective and accessible compared to PET, making it suitable for routine clinical use and large‐scale studies [5]. Moreover, the higher magnetic field strength of ASL MRI at 3T improves the signal‐to‐noise ratio and spatial resolution, enabling more precise detection of CBF changes in critical brain regions like the hippocampus and occipital cortex.

Nevertheless, ASL MRI's sensitivity to motion and its reliance on the scanner's magnetic field strength are limitations that can affect the accuracy and reproducibility of the results [1].

Other limitations include variability in perfusion measurements across different scanner models and the absence of standardization in acquisition protocols [5, 20].

### 4.6 ASL Sequence

Optimizing sequences is also essential. Liu et al. demonstrated that a shorter post‐label delay of 1.5 s on CBF measurements in AD patients using 3D ASL MRI was more effective in detecting hypoperfusion in key brain regions, such as the PCC and precuneus, which are commonly affected in AD [28].

The study by Kilroy et al. compares two types of ASL MRI techniques: 2D echo planar imaging pCASL, which is a more traditional approach that acquires images slice by slice, quicker but often more prone to motion artifacts and with lower spatial resolution, and 3D GRASE pCASL, which captures the entire brain volume at once, offering higher spatial resolution and better signal‐to‐noise ratio [30]. The study found that 3D GRASE pCASL provided more reliable and consistent CBF measurements.

### 4.7 Artificial Intelligence (AI)

AI, machine learning (ML), and deep learning (DL) could be valuable tools in enhancing the diagnostic utility of ASL MRI.

As reported by Collij et al., ML applied to ASL perfusion maps can accurately classify AD, differentiate it from subjective cognitive decline, and also predict conversion from MCI to AD. This underscores the value of these technologies in early diagnosis and improved clinical decision‐making [31].

Zhang et al. instead demonstrated that applying DL‐based denoising techniques to ASL MRI significantly enhances the sensitivity for detecting AD‐related hypoperfusion patterns, thanks to an improved contrast‐to‐noise ratio and image quality, particularly in the precuneus and parietal regions, which are critical for AD diagnosis [5].

## 5 Conclusion

This systematic review underscores the utility of ASL and related imaging techniques in detecting and characterizing CBF changes in AD, particularly using 3T MRI. The consistent finding of reduced CBF in key brain regions among AD patients, along with emerging evidence of hypoperfusion and associated structural changes, suggests that this imaging modality holds significant promise for both diagnostic and therapeutic monitoring in AD. Further research with more detailed demographic reporting and larger sample sizes is recommended to validate these findings and explore their clinical applications.

## Conflicts of Interest

The authors declare no conflicts of interest.
